# Economic analysis of sugammadex versus neostigmine for reversal of neuromuscular blockade for laparoscopic surgery in China

**DOI:** 10.1186/s13561-020-00292-x

**Published:** 2020-11-14

**Authors:** Maodong Ren, Ying Wang, Yan Luo, Jia Fang, Yongji Lu, Jianwei Xuan

**Affiliations:** 1 Shanghai Centennial Scientific Co. Ltd, Shanghai, China; 2 Department of Anesthesia and Pain Management, Shanghai Jiaotong University School of Medicine Ruijin Hospital, Shanghai, China; 3 Sun Yat-sen University, Guangzhou, China

**Keywords:** Sugammadex, Neostigmine, Residual neuromuscular blockade, Economic analysis

## Abstract

**Background:**

Neuromuscular blockade and pneumoperitoneum (PP) are important factors to ensure successful laparoscopic surgery. However, residual neuromuscular blockade (rNMB) and PP are associated with many unfavorable complications. The aim of this study is to compare the cost-effectiveness of using sugammadex versus neostigmine in laparoscopic surgery in China.

**Methods:**

A decision tree model was developed with a time horizon based on laparoscopic surgery related hospitalization duration. 2000 patients using sugammadex or neostigmine were simulated within the model. The model outcomes included incidence of rNMB and PP related complications and their treatment costs. Data on clinical efficacy, safety and cost were collected from published literature and interviews of physicians.

**Results:**

The model projected that treatment with sugammadex instead of neostigmine would lead to 673 fewer total complications, including rNMB/PP related complications, hospitalization, and other AEs (621 events versus 1294 events, respectively). Use of sugammadex was associated with an incremental medication cost of ¥1,360,410. However, 93.6% of the increased medication cost can be off-set by the reduced costs attributable to treatment of rNMB related complications, PP related complications, hospitalization and other adverse events in sugammadex group. In aggregate, the sugammadex group incurred an incremental cost of ¥86,610 to prevent 673 complications, (¥128.56 per one rNMB/PP related complications prevention). One-way sensitivity analysis confirmed the robustness of the model.

**Conclusions:**

Use of sugammadex in replacement of neostigmine would result in significantly lower rNMB/PP related complications but at a substantially higher medication cost. Upon accounting for the costs associated with treatment of rNMB/PP related complications, 93.6% of medication cost is projected to be offset. In balance, sugammadex appears to offer good value for reversal of neuromuscular blockade for laparoscopic surgery in China.

## Background

Neuromuscular blockade (NMB) and pneumoperitoneum (PP) are important factors to ensure successful laparoscopic surgery. However, in clinical practice, residual neuromuscular blockade (rNMB) may occur as long as neuromuscular blockade drugs are used. Clinically, rNMB is defined as the train-of-four ratio (TOFr) < 0.9 [1]. A real-world study in the Chinese population showed that the incidence of rNMB after tracheal extubation and arrival at post-anesthesia care unit (PACU) were 57.8 and 45.2%, respectively [2]. Many potential complications can be caused by rNMB, such as: hypoxemia, airway obstruction, muscle weakness, pulmonary related complications due to ineffective cough, pharyngeal dysfunction, etc [3]

To provide a good surgical condition, PP must be made in laparoscopic surgery. But high-pressure PP can lead to potential side effects, such as nausea/vomiting, shoulder pain and others [4, 5]. Deep NMB (post-tetanic count = 1 or 2) and low-pressure PP (less than 10 mmHg) are recommended in laparoscopic surgery by Chinese clinical guidelines [3]. It not only can improve laparoscopic surgical condition, but also can reduce PP side effects [3]. In Chinese clinical practice, most physicians perform laparoscopic surgeries in patients outside of deep NMB state due to a lack of deep NMB reversal drugs.

The results from controlled clinical trials have demonstrated that sugammadex can quickly reverse or moderate deep NMB compared with neostigmine [6, 7]. It can potentially reduce rNMB risk and at the same time, maintain a desirable deep NMB state during laparoscopic surgery. However, the high cost of sugammadex, prevents it from being used as a standard neuromuscular reversal drug. There is currently no comprehensive economic assessment comparing the cost-effectiveness between sugammadex and neostigmine in China. A Health Technology Assessment (HTA) performed by National Institute for Health Research (NIHR) in the United Kingdom used cost minimization analysis to evaluate the reduction in recovery time by using sugammadex and the value of each minute of recovery time saved [8]. Sugammadex appeared cost-effective for the routine reversal of rocuronium-induced moderate blockade at current listed price in UK (2 ml × 10 vials, £ 596.4; 5 ml × 10 vials, £ s1491.00) if all reductions in recovery time associated with sugammadex were achieved in the operating room. However, the use of sugammadex was not cost-effective if all reductions in recovery time were achieved in the recovery room instead. The cost-effectiveness of sugammadex is therefore highly dependent on the setting in which it is administered.

There were a number of limitations of this study. Data inputs were derived from systematic review of various randomized controlled trials of sugammadex, so comparators were quite diverse. Relevant outcomes in resources or costs used were also not investigated or reported in the trials. In this study, the cost-effectiveness between sugammadex and neostigmine will be assessed under China real-world scenario by quantifying rNMB and PP related complications and their treatment costs in laparoscopic surgery.

## Methods

A decision tree model was developed within Microsoft Excel from a payer’s perspective to analyze the incremental cost of avoiding one rNMB/PP complications by using sugammadex versus neostigmine for reversal of neuromuscular blockade for laparoscopic surgery. The model had a time horizon of laparoscopic surgery related hospitalization duration. Model inputs included the efficacy and safety data as reported in clinical trials and healthcare resource use and cost data obtained by physician interviews.

### Model structure

**Fig. 1:**
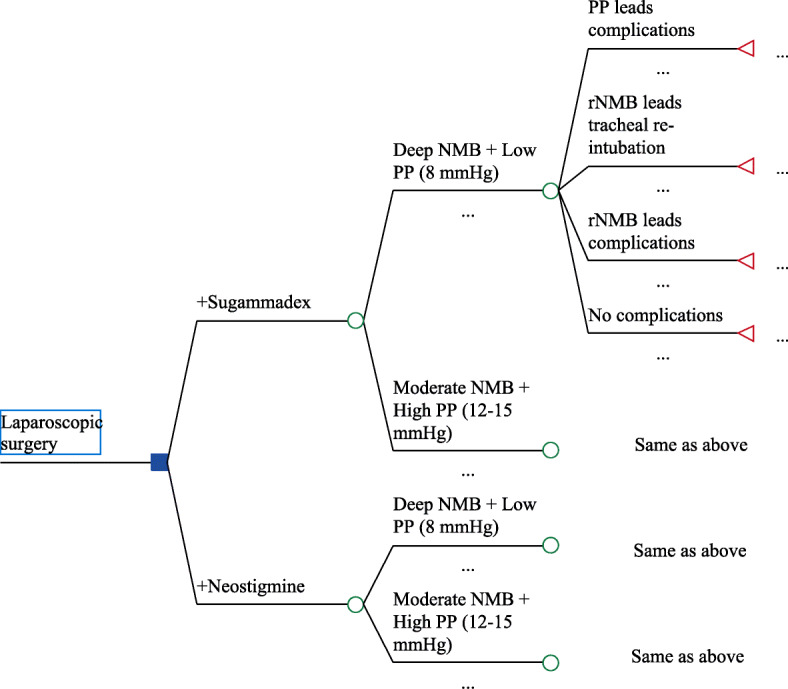
The decision tree model structure

The structure of decision tree model which simulated outcomes of interest from 2000 patients receiving laparoscopic surgeries is shown in Fig. 1. The initial decision node was whether the patients used sugammadex or neostigmine for reversal of NMB. To ensure desirable surgical condition, physicians need to choose one surgical strategy: deep NMB plus low PP pressure (8 mmHg) or moderate NMB plus high PP pressure (12–15 mmHg). The probabilities of different surgical strategies were affected by choice of sugammadex or neostigmine. Finally, the model endpoint was rNMB / PP associated complications.

### Clinical data

The clinical data were mainly obtained from published literature. rNMB and PP were the main sources of complications. The incidence rate of rNMB for sugammadex and neostigmine was 0 and 44.5%, respectively [9]. The tracheal re-intubation rate was 1.06 and 0% in rNMB and no rNMB situation, respectively [10]. The rNMB / PP related complication rate and unit cost are presented in Tables 1 & 2. In addition, adverse events (AEs) occurred for ≥5% subjects in sugammadex or neostigmine group as reported in the clinical trial were considered in the model with exception of procedure nausea or vomiting per input from the key opinion leader (KOL) [14].

**Table 1:** rNMB related complications related inputs

	rNMB related complications			rNMB related complications cost	
	rNMB	No rNMB	Sources	Cost (Yuan)	Sources
Pharyngeal dysfunction	28.00%	13.00%	[11]	138.04	KOL
Upper airway obstruction^a^	8.42%	1.68%	KOL & [12]	235.62	KOL
Lower airway obstruction^a^	1.58%	0.32%	KOL & [12]	1179.61	KOL
Mild-moderate hypoxemia	23.00%	4.00%	[12]	118.92	KOL
Severe hypoxemia	7.00%	1.00%	[12]	777.86	KOL
Respiratory failure	8.00%	1.00%	[12]	27,706.57	KOL & [13]
Muscular weakness	16.00%	1.00%	[12]	1269.89	KOL

^a^Note: the literatures reported the incidence rate of airway obstruction in rNMB and no rNMB is 10 and 2% respectively. The KOL reported the percentage of upper airway obstruction and lower airway obstruction are accounted for 84.24 and 15.76% in airway obstruction. Hence, the incidence rate of upper and lower airway obstruction can be calculated

**Table 2:** PP related complications related inputs

	PP related complications			PP related complications cost	
	8 mmHg	12–15 mmHg	Sources	Cost (Yuan)	Sources
Shoulder pain	23.30%	73.30%	[4]	105.15	KOL
Nausea & Vomiting	0.00%	25.00%	[5]	107.81	KOL

### Resource use & cost data

The probabilities of different surgical strategies were obtained from KOL interviews (Table 3). The KOL interviews included 30 experienced anesthesiologists from 10 different hospitals (tier 3) located in Beijing, Shanghai, Nanjing, Chengdu, Guangzhou, and Wuhan in China.

**Table 3:** Probabilities of different surgical strategies

	Surgical strategies	Probability	Source
Sugammadex	Deep NMB + Low PP pressure (8 mmHg)	77.67%	KOL
	Moderate NMB + High PP pressure (12–15 mmHg)	22.33%	
Neostigmine	Deep NMB + Low PP pressure (8 mmHg)	30.00%	
	Moderate NMB + High PP pressure (12–15 mmHg)	70.00%	

The high-pressure PP (12–15 mmHg) was associated with an extra 0.27 hospitalization days compared with low-pressure PP (8 mmHg) [15], and the average cost of laparoscopic surgery was ¥1524.18 per day [16].

For each laparoscopic surgery, costs for sugammadex and neostigmine were estimated to be ¥1380 and ¥6.53, respectively based on the average bidding price from: Yaozhi website (https://db.yaozh.com/).

The costs of rNMB / PP related complications were estimated from KOL interviews and published literature (Tables 1 & 2). AEs treatment costs were obtained from KOL so was the cost of tracheal re-intubation (¥1050).

### Sensitivity analysis

We performed one-way sensitivity analysis to investigate the robustness of the model by varying up or down 10% of base-case values for all model parameters.

## Results

### Base-case analysis

With 2000 patients simulated, the model projected that treatment with sugammadex instead of neostigmine would lead to 673 fewer total complications, including rNMB / PP related complications, hospitalization, and other AEs (621 events versus 1294 events, respectively). Although the sugammadex group was associated with an incremental medication cost of ¥1,360,410, the costs related to treatment of rNMB related complications, PP related complications, hospitalization and other adverse events in sugammadex group were all projected to be lower than those in the neostigmine group, leading to net cost-savings of ¥1,006,503, ¥37,906, ¥33,228, ¥196,162, respectively (Table 4). In aggregate, the sugammadex group incurred an incremental cost of ¥86,610 to prevent 673 complications, resulting in an incremental cost of ¥128.56 to avoid one rNMB / PP related complication.

**Table 4:** The summary of the model results

Item	Sugammadex	Neostigmine	Difference
Medication cost	¥ 1380,000	¥ 19,590	¥ 1,360,410
rNMB related complications cost	¥ 327,933	¥ 1,334,436	¥ -1,006,503
PP related complications cost	¥ 44,407	¥ 86,900	¥ -37,906
Hospitalization cost	¥ 91,908	¥ 288,071	¥ -196,162
Other adverse events cost	¥ 145,641	¥ 178,869	¥ -33,228
Total cost	¥ 1,987,739	¥ 1,901,129	¥ 86,610
Total complications frequency	621	1294	−673

Of the simulated patient population, shoulder pain, pharyngeal disorders, and postoperative nausea and vomiting ranked the top three complications at 48.45, 17.07 and 12.06% respectively.

### One-way sensitivity analysis

**Fig. 2:**
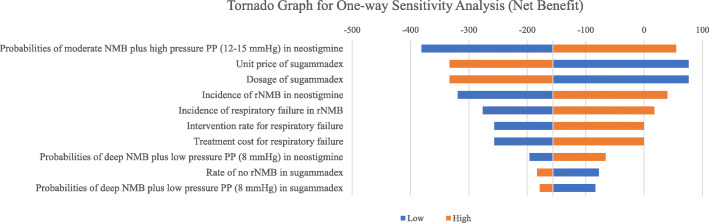
Tornado diagram for top ten populated parameters within the model

A tornado diagram was developed to illustrate the top ten populated parameters regarding the cost of avoiding one rNMB / PP related complication for the sugammadex group compared with that of the neostigmine group (Fig. 2). The most influential parameter was the probabilities of moderate NMB plus high pressure PP (12–15 mmHg) in neostigmine group, followed by unit price of sugammadex, dosage of sugammadex, incidence rate of rNMB in the neostigmine group, incidence of respiratory failure, treatment cost for respiratory failure, probabilities of deep NMB plus low pressure PP in neostigmine, the rate of no rNMB in the sugammadex group, and probabilities of deep NMB plus low pressure PP in the sugammadex group.

## Discussion

Our study indicated that use of sugammadex instead of neostigmine for reversal of neuromuscular blockade in laparoscopic surgery would result in significantly lower rNMB / PP related complications at a much higher incremental medication cost. However, 93.6% of the increased medication cost can be off-set by the reduced costs attributable to treatment of rNMB related complications, PP related complications, hospitalization and other adverse events in sugammadex group. Net to net, our model projected that use of sugammadex would lead to an incremental cost of ¥128.56 to prevent one rNMB / PP related complication. It is worth noting that we only focused on the potential benefits of sugammadex with regarding to reduction of complications associated with rNMB or PP. In fact, another important clinical benefit of sugammadex is a significantly shorter time required for reversal of NMB [17], which could lead to shorter operation time and improving the efficiency of the operating room utilization.

The findings from our study are consistent with the results of a controlled clinical trial which compared sugammadex with neostigmine in patients undergoing surgery for the treatment of obstructive sleep apnea [18]. The trial showed that patients receiving sugammadex had shorter time to TOF 0.9 and operating room time. More importantly, compared neostigmine, sugammadex decreased the incidence of post-operative respiratory complications and related costs. Similar findings were also observed with real-world data. De et al. conducted a retrospective analysis from records of morbidly obese patients undergoing elective laparoscopic bariatric surgery in which sugammadex or neostigmine was used to reverse NMB. The analysis showed that, compared with neostigmine, use of sugammadex for 50 laparoscopic bariatric surgeries resulted in a total savings of 19.4 h (average 23.3 min per laparoscopic bariatric surgery). It was projected that an additional 12 laparoscopic gastric sleeve resections can be performed with the time saved by using sugammadex [17].

There are several limitations with our study. We did not include indirect costs and may have underestimated the benefits of sugammadex due to lack of relevant research on operating room and labor costs in China. The study also assumed that the incidence of rNMB / PP related complications in laparoscopic surgery for different diseases was the same. However, via the interviews, more than 50% of KOLs believed that the incidence of rNMB / PP related complications in laparoscopic surgery for different diseases was the same so we deemed this to be an appropriate assumption. Our model’s time horizon of laparoscopic surgery related hospitalization and the results may also not be representative to open general anesthesia surgery although factors associated with rNMB are mainly related to the operation duration, anesthetic medications, anesthesia methods, post-operative monitoring and the choice of NMB reversal medications [10]. Finally, the cost data of this study were estimated based on input from 30 anesthesiologists, representing experiences from tertiary teaching hospitals in major metropolitan cities in China. In the future, a nation level study for cost should be carried out to improve the quality of data.

## Conclusion

Compared with neostigmine, use of sugammadex for reversal of neuromuscular blockade for laparoscopic surgery is expected to result in better clinical outcomes by reducing rNMB / PP related complications. Although sugammadex is much more expensive in anesthesia practice than neostigmine, over 90% of medication cost can be offset by reduced costs associated with treatment of rNMB / PP related complications. In balance, sugammadex appears to offer good value for reversal of neuromuscular blockade for laparoscopic surgery in China.

## Data Availability

Data on clinical efficacy, safety and cost were collected from published literature and interviews of physicians.

## Abbreviations

AE: Adverse Event
HTA: Health Technology Assessment
KOL: Key Opinion Leader
NIHR: National Institute for Health Research
NMB: Neuromuscular Blockade
PACU: Post-Anesthesia Care Unit
PP: Pneumoperitoneum
rNMB: residual Neuromuscular Blockade
TOFr: Train-of-Four ratio

## References

[1] Murphy GS, Brull SJ (2010). Residual neuromuscular block: lessons unlearned. Part I: definitions, incidence, and adverse physiologic effects of residual neuromuscular block. Anesth Analg.

[2] Buwei Y (2015). Incidence of postoperative residual neuromuscular blockade after general anesthesia: a prospective, multicenter, anesthetists-blind, observational study. Curr Med Res Opin.

[3] Wu, X. Expert Consensus on Rational Drug Use in Clinical Practice for Neuromuscular Blockade (2017 Edition). Beijing: People's Medical Publishing House (PMPH)., 2017.

[4] Sroussi J (2017). Low pressure gynecological laparoscopy (7 mmHg) with AirSeal1 system versus a standard insufflation (15 mmHg): a pilot study in 60 patients.

[5] Hu J, Zhang R, Bao H (2012). Impact of different intra-abdominal pressure on hemodynamic changes monitored by FloTrac/Vigileo in laparoscopic gynecological surgery patient. J Clin Anesth.

[6] Wu X (2014). Rocuronium blockade reversal with sugammadex vs. neostigmine: randomized study in Chinese and Caucasian subjects. BMC Anesthesiol.

[7] Yu B, Wang X, Helbo-Hansen HS, Huang WQ, Askeland B (2014). Sugammadex 4.0 mg kg–1 reversal of deep Rocuronium-induced neuromuscular blockade: a multicenter study in Chinese and Caucasian patients. J Anesth Clin Res.

[8] Chambers D, Paulden M, Paton F, Heirs M, Duffy S, Craig D, et al. Sugammadex for the reversal of muscle relaxation in general anaesthesia: a systematic review and economic assessment. Health Technol Assess. 2010;14(39):1-211. 10.3310/hta14390 20688009

[9] Carron M, Baratto F, Zarantonello F (2016). Sugammadex for reversal of neuromuscular blockade: a retrospective analysis of clinical outcomes and cost-effectiveness in a single center. Clinicoecon Outcomes Res Ceor.

[10] Errando CL, Garutti I, Mazzinari G (2016). Residual neuromuscular blockade in the postanesthesia care unit. Observational cross-sectional study of a multicenter cohort. Minerva Anestesiol.

[11] Sundman E, Witt H, Olsson R, Ekberg O, Kuylenstierna R, Eriksson LI (2000). The incidence and mechanisms of pharyngeal and upper esophageal dysfunction in partially paralyzed humans. Pharyngeal videoradiography and simultaneous manometry after atracurium. Anesthesiology.

[12] Norton M, Xará D, Parente D (2013). Residual neuromuscular block as a risk factor for critical respiratory events in the post anesthesia care unit. Rev Esp Anestesiol Reanim.

[13] Wang Y, Zhang Q, Li L, Wang S (2015). Length of stay in intensive care unit and total cost with midazolam combined with fentanyl among intubated patients with type II AECOPD respiratory failure. China Pract Med.

[14] Wu X, Oerding H, Liu J (2014). Rocuronium blockade reversal with sugammadex vs. neostigmine: randomized study in Chinese and Caucasian subjects[J]. BMC Anesthesiol.

[15] Hua J, Gong J, Yao L (2014). Low-pressure versus standard-pressure pneumoperitoneum for laparoscopic cholecystectomy: a systematic review and meta-analysis. Am J Surg.

[16] Jia X, Dong Y (2010). Comparative analysis of length of stay and total cost between traditional surgical approach and laparoscopy. China Foreign Med Treat.

[17] De RE (2016). The use of sugammadex for bariatric surgery: analysis of recovery time from neuromuscular blockade and possible economic impact. Clinicoecon Outcomes Res Ceor.

[18] Ünal DY, Baran İ, Mutlu M (2015). Comparison of Sugammadex versus Neostigmine Costs and Respiratory Complications in Patients with Obstructive Sleep Apnoea. Turk J Anaesthesiol Reanim.

